# Brain biopsy in patients with CLIPPERS syndrome: why and when

**DOI:** 10.1177/17562864211062821

**Published:** 2022-01-30

**Authors:** Imke Galazky, Lars Büntjen, Jürgen Voges, I. Erol Sandalcioglu, Christian Mawrin, Aiden Haghikia

**Affiliations:** Department of Neurology, Otto-von-Guericke-University Magdeburg, Leipziger Straße 44, 39120 Magdeburg, Germany; Department of Stereotactic Neurosurgery, Otto-von-Guericke University Magdeburg, Magdeburg, Germany; Department of Stereotactic Neurosurgery, Otto-von-Guericke University Magdeburg, Magdeburg, Germany; German Center for Neurodegenerative Disease (DZNE), Bonn, Germany; Department of Neurosurgery, Otto-von-Guericke University Magdeburg, Magdeburg, Germany; Institute of Neuropathology, Otto-von-Guericke University Magdeburg, Magdeburg, Germany; Department of Neurology, Otto-von-Guericke-University Magdeburg, Leipziger Straße 44, 39120 Magdeburg, Germany; German Center for Neurodegenerative Disease (DZNE), Bonn, Germany

**Keywords:** brain biopsy, CLIPPERS, CNS lymphoma, mimic, MRI

## Abstract

CLIPPERS (chronic lymphocytic inflammation with pontine perivascular enhancement responsive to steroids) is an inflammatory disorder of the central nervous system (CNS), predominantly involving the brainstem with a characteristic magnetic resonance imaging (MRI) appearance and clinical and radiological responsiveness to glucocorticosteroids. Yet diagnostic biomarkers are missing and other immune-mediated, (para-) infectious and malignant causes mimic CLIPPERS-like MRI presentations. We report the case of a 51-year-old male patient with CLIPPERS who repeatedly responded well to high-dose corticosteroids. After 7 months, however, treatment failed, and he had a biopsy-confirmed diagnosis of a CNS B-cell lymphoma. Clinical and MRI signs of CLIPPERS include a wide spectrum of differential diagnoses which often arise only later during the course of disease. Similar to the case presented here, delayed diagnosis and specific therapy may contribute to an unfavorable outcome. Hence, we propose that in the absence of other diagnostic markers, brain biopsy should be performed as early as possible in CLIPPERS patients.

## Introduction

Chronic lymphocytic inflammation with pontine perivascular enhancement responsive to steroids (CLIPPERS) describes an inflammatory disease of the central nervous system (CNS) predominantly detected within the brainstem characterized by typical nodular and punctuate contrast enhancement and a strong therapeutic responsiveness to glucocorticosteroids.^1^ Histopathological hallmarks of CLIPPERS lesions are perivascular, predominantly CD3+ T-cell infiltrates.

Since the first description of CLIPPERS, various similar cases have been reported.^2,3^ The re-assessment of a series of 24 histologically examined patients with an unspecific inflammatory demyelinating CNS disease revealed a CLIPPERS syndrome in 12.5% of all cases assessed.^4^

Atypical findings, so-called ‘red flags’, which deem the diagnosis of CLIPPERS syndrome unlikely, include the following:

- Inadequate response to glucocorticosteroid therapy at onset or during the course of disease;
- Absence of common symptoms of CLIPPERS syndrome such as dysarthria and ataxia, as well as additional clinical symptoms, such as fever, B symptoms, extracerebral organ manifestations (e.g. arthritis, uveitis, sicca syndrome, lymphadenopathy, and others), and meningism;
- MRI findings without brainstem pathology (absence of this constellation is not associated with CLIPPERS), pontine lesions with necrosis (indicative of primary CNS lymphoma), and marked space-occupying lesions (CNS tumors in general);
- Marked CSF pleocytosis (>100/μl) or abnormal cells.

In 2012, the diagnostic criteria of CLIPPERS syndrome were expanded based on the core characteristics including (1) clinical and (2) radiological findings, as well as (3) response to glucocorticosteroids, and (4) the presence of histopathological criteria. It was postulated that in the presence of criteria 1–3 and by exclusion of alternative causes by detailed noninvasive diagnostics CLIPPERS can be diagnosed with a high probability.^5^

In addition to the initial reports, extension of CLIPPERS lesions into adjacent CNS structures has been described both caudally to the medulla oblongata, the cervico-thoracic spinal cord, and also rostrally to the midbrain. Supratentorial regions such as the thalami, internal capsule, basal ganglia, corpus callosum, and subcortical white matter can also be affected.^1,2,6–10^ Even in periods of clinical remission, supratentorial lesions and perivascular abnormalities with contrast-enhancing lesions centered around the small venous vessels were detectable on high-resolution 7.0 T magnetic resonance imaging (MRI).^11^

## Case

**Figure 1.**
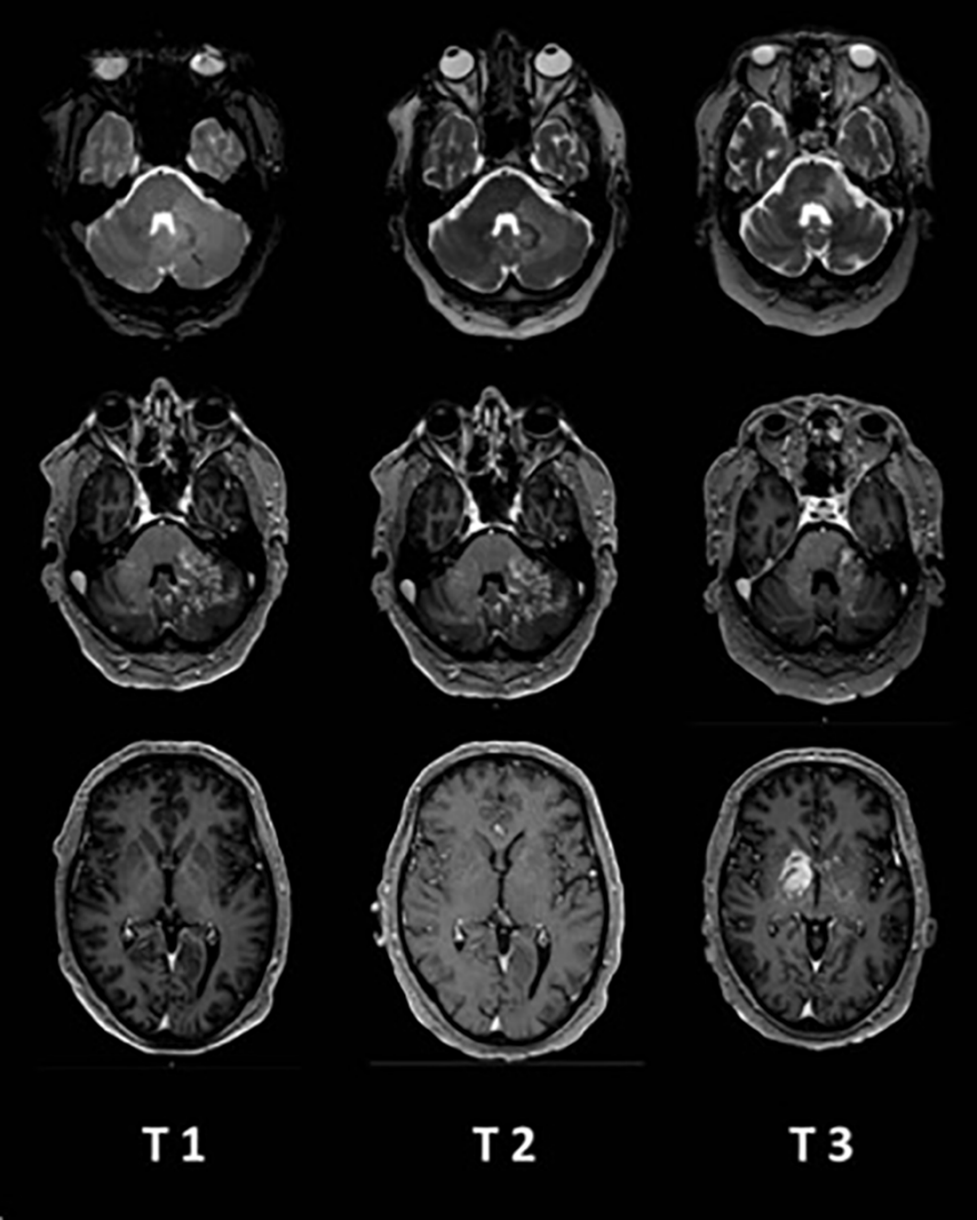
Development of MRI patterns from the time of initial symptoms (T1) to 4 weeks (T2) and 6 months (T3). The upper row shows T2-weighted images of the brainstem, the middle row shows gadolinium enhancement of the brainstem, and the lower row shows gadolinium enhancement of the supratentorial region. MRI, magnetic resonance tomography.

We report on a 51-year-old patient who progressively developed left-sided ataxia with gait unsteadiness, dysarthria, diplopia, and mild right facial paresis. MRI examination (Figure 1, T1) showed extensive bilateral cerebellar patchy and striated gadolinium enhancement, most likely inflammatory of origin. Cerebrospinal fluid (CSF) diagnosis revealed pleocytosis of 17/µl (lymphomonocytosis) with a total protein of 548 mg/l. Oligoclonal bands were negative. Extended diagnosis excluded infectious pathogens, vasculitis, paraneoplastic disease, and sarcoidosis. The image morphology was described compatible with a CLIPPERS syndrome; therefore, a therapy with i.v. methylprednisolone 1 g/d for 3 days was performed. Under this therapy regimen, the symptoms improved. At this point, we decided to postpone a possible bioptic workup after a stringent risk–benefit assessment.

About 4 weeks later, there was a clinical relapse of the ataxia with increasing stance and gait unsteadiness and worsening of diplopia. MRI (Figure 1, T2) showed a slight regression of the small-spotted and striated barrier disturbances in the left cerebellar hemisphere and in the left middle cerebellar peduncle compared to the previous examination. Contrast enhancement in the cerebellar vermis was constant and discretely progressive in the right cerebellar hemisphere and middle cerebellar peduncle. Concomitant T2 changes in both cerebellar hemispheres and in the middle cerebellar peduncles and in the cerebellar vermis were also mildly progressive. Isolated punctate hyperintensities after gadolinium administration in the brainstem appeared and were interpreted as vascular gating. Supratentorial lesions were not detectable. CSF pleocytosis was discretely regressive (6/µl; 91% lymphocytes, 9% monocytes) and total protein was 771 mg/l. Repeated therapy with i.v. methylprednisolone 1 g/d for 5 days improved the clinical symptoms markedly.

Another 12 weeks later, the above described symptomatology worsened again. After repeated cortisone pulse therapy, a significant clinical improvement was documented. In addition, methotrexate was initiated as a basic immunotherapy.

Approximately 6 months after the onset of the disease, a massive clinical deterioration occurred with an acceleration of ataxia, dysarthria, and dysphagia, as well as the occurrence of a tetraparesis leading to almost complete dependence on nursing care. Repeated CSF analysis showed lympho-monocytic pleocytosis, and identical oligoclonal bands in CSF and serum were detectable. Flow cytometry revealed predominantly T cells (77.8% of all CD45-positive cells) and no evidence of a monotypic B cell population. MRI (Figure 1, T3) demonstrated increasing and new contrast enhancement with expansion into the right thalamus and pallidum.

**Figure 2.**
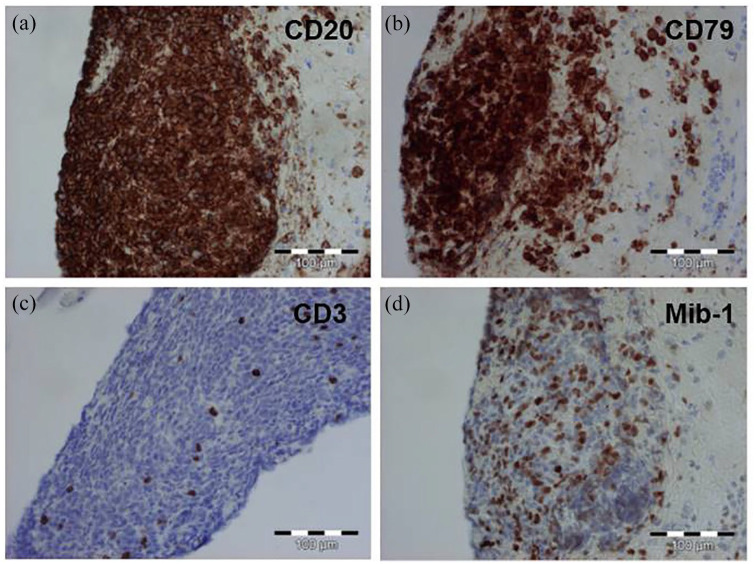
Neuropathological features of stereotactic brain biopsy. Dense infiltration of CD20 (a) and CD79a (b) positive B cells. (c) CD3 positive T cells are sparse. (d) Enhanced proliferation activity of the lesion as detected by the proliferation marker Mib-1.

A stereotactic biopsy from the right pallidum was performed at this time. The histopathology revealed dense infiltrates of CD20- and CD79a-positive B lymphocytes with high proliferation activity (Figure 2), which led to the diagnosis of a highly malignant B-cell lymphoma. Advanced staging with thoracic and abdominal computed tomography (CT) scan, and bone marrow analysis revealed no evidence of extracerebral manifestations of the lymphoma.

During further hemato-oncological treatment with rituximab and methotrexate, the patient died 10 months after the onset of disease.

Informed consent regarding publication of the individual medical data was received and written by the kin of the reported patient.

## Discussion

Meanwhile, a large number of cases with differential diagnoses showing CLIPPERS-like neuroradiological features have been described in the literature (Table 1). In a PubMed search including cases from 2010 to 2020 with the keyword ‘CLIPPERS’, 41 of 97 entries referred to case descriptions of CLIPPERS mimics. In two case series, alternative diagnoses occurred in 13 of 42 (30%), and 12 of 23 (52%), respectively, patients with an initial diagnosis of CLIPPERS syndrome.^12,13^ Seven of 42 patients (16%) were diagnosed with CNS lymphoma during follow-up.^12^ Proton-weighted MR spectroscopy was proposed as potentially helpful in differentiating these cases from CLIPPERS.^14^

**Table 1.** CLIPPERS mimics.

Disease entity	Diagnosis	N	Age/sex	Biopsy	Source
Malignant diseases	Glioma	1	46/M^1^	Brain	Jones *et al.*^15^
	Primary CNS lymphoma	1	57/M	Brain	De Graaff *et al.*^16^
		1	33/M	2 x brain	Limousin *et al.*^14^
		1	74/M	Brain and autopsy	Lin *et al.*^17^
		1	58/M	2 x brain	Taieb *et al.*^18^
		1	40/M	2 x brain	Link *et al.*^19^
	EBV-positive B cell lymphoma	1	33/M	Brain	Ahn *et al.*^20^
	Systemic T cell lymphoma	1	42/F	No	Nakamura *et al.*^21^
	Following Hodgkin lymphoma	1	31/M	Brain	Mashima *et al.*^22^
	Limbic encephalitis	1	37/F	No	Ohta *et al.*^23^
	Primary lymphomatoid CNS granulomatosis	1	51/F	Brain	Wang *et al.*^24^
		1	31/F	Brain	Tian *et al.*^25^
	Cutaneous T cell lymphocytosis	1	59/F	Skin	Smith *et al.*^26^
(Para-) infectious	Infection with hepatitis B	1	34/M	No	Weng *et al.*^27^
	Infection with Epstein–Barr virus	1	37/M	Brain	Ma *et al.*^28^
	Post influenza vaccination	1	80/M	Brain	Hillesheim *et al.*^29^
Immune mediated	Angiitis	1	45/F	Brain	Buttmann *et al.*^30^
	Lymphohistiocytosis	1	38/M	Lymph node	Li *et al.*^31^
	Cutaneous sclerodermia	1	26/F	No	Anand *et al.*^32^
	Erdheim Chester histiocytosis	1	52/M	Kidney	Berkman *et al.*^33^
	Hashimoto thyroiditis	1	59/F	No	Yiannopoulou *et al.*^34^
	HLA B27 associated uveitis	1	15/M	No	Crowell *et al.*^35^
	Anti-IgLON5-syndrome	1	76/M	Brain	Rössling *et al.*^36^
	Association with MOG antibodies	1	43/M	No	Berzero *et al.*^37^
		1	36/F	No	Symmonds *et al.*^38^
	Multiple sclerosis	1	28/F	No	Ferreira *et al.*^39^
		1	61/F	Brain	Ortega *et al.*^40^

CLIPPERS, chronic lymphocytic inflammation with pontine perivascular enhancement responsive to steroids; CNS, central nervous system; EBV, Epstein–Barr virus; HLA, human leukocyte antigen; MOG, myelin oligodendrocyte glycoprotein.

Based on these observations, CLIPPERS is considered an initial clinical syndrome with multiple underlying causes that may represent the precursor of a malignoma.^18,41^ In two CLIPPERS patients, immunotyping of perivascular lymphocyte infiltrates revealed CD20-positive B lymphocytes. This was interpreted an initial sign of possible conversion to a lymphoma.^42^

Parainfectious, immune-mediated, and paraneoplastic CLIPPERS mimics can be detected by extended laboratory diagnostics. However, in the absence of defined diagnostic biomarkers, neuropathological examination of the affected brain areas should be considered.

Recommendations for brain biopsy – provided a sufficiently safe procedure is warranted – are based on the high diagnostic gain with reasonably low morbidity from a biopsy in the posterior fossa.^13^ However, repeated biopsy may be necessary, that is, in some patients the diagnosis of lymphoma could only be confirmed histologically by a second biopsy.^12^ Brain biopsy has been recommended if (a) the gadolinium-enhancing lesion is unilateral or (b) in the case of larger lesions (>9 mm,^12^ >3 mm^13^). Indeed, at the first clinical contact, MRI of the presented patient showed asymmetric and patchy gadolinium enhancements and also T2 hyperintensities exceeding in size the contrast-enhancing lesions, which are not typical for CLIPPERS. However, after a thorough risk–benefit assessment, brain biopsy was considered too risky due to the eloquent localization of brain lesions in the brainstem. A biopsy is not always feasible,^13^ and some authors even argued that histological confirmation of the diagnosis is dispensable in the absence of atypical features. They claim that (1) a clear response to corticosteroids and progressive atrophy of the affected brain region in the course of the disease deem a malignant process unlikely, or that (2) a small sample size may lead to false-negative results.^1,9,43^ However, a response to corticosteroids does not rule out the presence of lymphoma. In addition, the histological decay of cerebral lymphomas after exposure to corticosteroids may mimic CLIPPERS on contrast-enhanced T1-weighted MRI. Furthermore, at the time of initial manifestation of clinical symptoms, information of the previous course of the disease is not available in the individual case.

A contrary source of criticism concerns the strategy that patients with primary CNS lymphomas receive corticosteroids – sometimes for months – for symptom control before biopsy is performed. This strategy can lead to distorted interpretation of diagnostic imaging and tissue probes unsuitable for correct diagnosis. In addition, these patients have a higher risk of complications such as diabetes and infections.^44^ A clinical risk assessment should weight the risk of taking a biopsy against the risk of missing the diagnosis allowing for qualified informed consent. A systematic review for the diagnostic value and safety of stereotactic biopsies for brainstem tumors of 1480 cases revealed over 96% of diagnostic success, 1.7% of permanent morbidity, and 0.9% mortality for these exceptionally difficult location.^45^ Likewise, in the case presented here, it is conceivable that a prolonged diagnostic workup would lead to delayed specific treatment. This underscores the need for early histopathological examination of suspicious brain lesions before it is legitimate to make the definitive diagnosis of CLIPPERS syndrome.

Based on our experience with the presented case and from implications by other case reports, showing that only brain biopsy leads to diagnosis, the authors come to the conclusion that brain biopsy should be performed as early as possible in case of clinical atypical presentation, when complementary examinations remain negative, especially in the presence of radiological atypical features.^12,13^ The decision to perform brain biopsy has to be made in a case-by-case manner after thorough risk–benefit assessment.
